# Sims: An interactive tool for geospatial matching and clustering

**DOI:** 10.1371/journal.pone.0344525

**Published:** 2026-04-08

**Authors:** Akram Zaytar, Girmaw Abebe Tadesse, Caleb Robinson, Eduardo G. Bendito, Medha Devare, Meklit Chernet, Gilles Q. Hacheme, Rahul Dodhia, Juan M. Lavista Ferres

**Affiliations:** 1 Microsoft AI for Good Research Lab, Nairobi, Kenya; 2 Microsoft AI for Good Research Lab, Redmond, Washington, United States of America; 3 International Institute of Tropical Agriculture, Nairobi, Kenya; 4 International Institute of Tropical Agriculture, Montpellier, Occitanie, France; Memorial Sloan Kettering Cancer Center, UNITED STATES OF AMERICA

## Abstract

Acquiring, processing, and visualizing geospatial data requires significant computing resources, especially for large spatio-temporal domains. This challenge hinders the rapid discovery of predictive features, which are essential for advancing geospatial modeling. To address this, we developed Similarity Search (Sims). This no-code web tool enables users to perform clustering and similarity search over defined regions of interest utilizing Google Earth Engine as its backend. Sims is designed to complement existing modeling tools by focusing on feature exploration rather than model creation. We demonstrate the utility of Sims through a case study analyzing simulated maize yield data in Rwanda, where we evaluate how different combinations of soil, weather, and agronomic features affect the clustering of yield response zones. Sims is open source and available at https://github.com/microsoft/Sims.

## Introduction

In geospatial analysis, acquiring, processing, and visualizing spatial data is time-consuming, particularly when dealing with datasets covering large spatio-temporal domains [1]. This makes it difficult to explore different layers and identify the most useful ones for a given task. Consequently, in many workflows, data products are selected based on domain expertise with limited exploratory analysis. Similar bottlenecks arise when building data processing pipelines. As the spatio-temporal domain expands, the exploration process slows due to longer acquisition and processing times, coupled with a lack of immediate visual feedback, making it difficult to iterate quickly. This creates a bottleneck: turning exploratory map views into model-ready feature stacks and candidate cohorts remains slow, ad hoc, and code-heavy.

Beyond these computational challenges, geospatial data presents unique statistical concerns [2]. Satellite imagery and other spatial data typically violate the independent and identically distributed assumption due to spatial autocorrelation—nearby locations tend to be more similar than distant ones. This spatial dependency complicates traditional methods for sampling, model validation, and experimental design, often leading to over-confident model evaluations or biased outcomes. While clustering techniques can help by grouping similar regions and enabling cluster-level sampling, the resource-intensive nature of geospatial data makes it difficult to efficiently implement these approaches in practice. Lastly, there are capacity hurdles that exclude those who may need to harness the power of geospatial data but are unable to do so because they do not have the required expertise.

Several tools have been developed to address these challenges. For instance, geemap [3] and Earth Map [4] provide interactive mapping and data exploration capabilities using Google Earth Engine (GEE) [5] as backend. However, mapping-centric tools stop short of modeling tasks—*feature matching* and *clustering* across multi-layer, time-varying stacks—where analysts need rapid, visual feedback to test hypotheses, compare feature sets, and curate training data.

To address this gap, we propose **Sims**, an interactive tool that simplifies geospatial feature discovery by integrating clustering and similarity search directly on GEE. While tools like geemap and Earth Map excel at visualizing and exploring individual layers, they lack built-in functionality for tasks central to modeling workflows—such as segmenting a region into homogeneous zones based on multiple features, or identifying areas with similar agro-ecological profiles to scale agricultural interventions. Embedding these tasks in an interactive app is compelling: the petabyte-scale GEE catalog and server-side reducers enable fast discovery over large areas without local downloads; immediate map updates tighten the experiment loop; and a no-code UI lowers the barrier for data scientists with limited Geographic Information System (GIS) tooling.

Sims adopts a user-centric design with interactive capabilities. For example, users select regions and time windows, choose feature layers, tune algorithms (e.g., number of clusters *k* and distance/cluster methods), preview results on the map, and export rasters/vectors all in real-time—accelerating the path from exploration to modeling without extensive coding or GIS expertise.

The specific objectives of this study were to:

- Introduce Sims, a tool that performs clustering and similarity search on geospatial data.
- Describe the workflow and design choices that enable interactive data visualization, alias-based feature construction, clustering and similarity search, and export of results.
- Demonstrate utility via a Rwanda maize-yield case study, assessing how variable groups influence yield-response zoning and statistical separation. Release an open-source implementation and discuss limitations and extensions to guide adoption and future work.

## Materials and methods

### Tool overview

**Fig 1:**
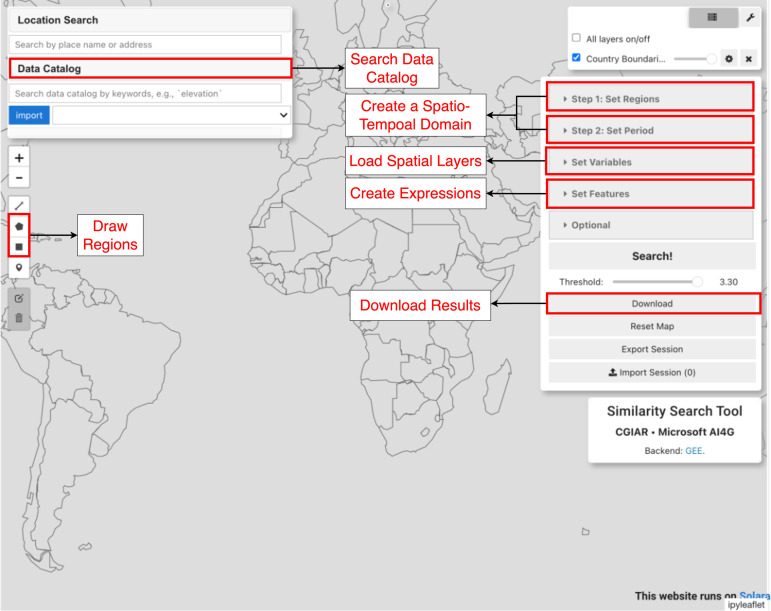
Overview of Sims. The interface includes functionalities such as searching the data catalog, drawing or uploading regions of interest, creating spatio-temporal domains, loading and visualizing layers from GEE, creating custom variable expressions, and downloading the resulting cluster or similarity maps.

Sims is built on top of *geemap* [3], *ipyleaflet* [6], and *Solara* [7]; these libraries respectively enable interactive mapping with GEE, dynamic geospatial widgets for Jupyter, and web deployment. Sims provides two main functionalities: *clustering* and *similarity search*. Clustering produces spatially similar sub-regions given a query region, time period, and variables of interest (i.e., a feature profile). For example, users can segment a country into homogeneous agricultural zones based on soil properties, rainfall, and temperature to guide region-specific interventions. In clustering, Sims groups pixels within the query region using GEE’s Weka clustering algorithm where users specify the number of clusters *k*. Unlike static zone maps, Sims provides on-demand, spatially and temporally relevant zones. For similarity search, Sims compares query and reference regions using configurable distance metrics (Euclidean, Manhattan, or Cosine distance), generating a heatmap that highlights areas most similar to the reference region. For example, given a reference region with known high crop productivity, users can identify other areas with similar agro-ecological conditions to scale successful farming practices. Both clustering and similarity search produce downloadable raster files for further analysis. Sims can be run locally or deployed with Solara [7]. Fig 1 shows the interface where users can draw or upload regions, choose periods, load and view layers from GEE, and create custom variable expressions (i.e., features).

To use the tool, users first define a spatio-temporal domain of interest by drawing geometries on the map or uploading vector files. For similarity search, two geometries are defined: the “query” and the “reference” regions. Next, a period is selected, and relevant GEE layers are loaded and visualized. Features can be created using GEE expressions (e.g., creating the feature ndvi=(nir-red)/(nir + red) from the nir and red aliases, where **NDVI** stands for Normalized Difference Vegetation Index, a widely used indicator of vegetation condition). Additionally, users may focus on specific land cover classes. Finally, the feature layers are resampled, stacked, and used to perform clustering or similarity search.

### Clustering

**Fig 2:**
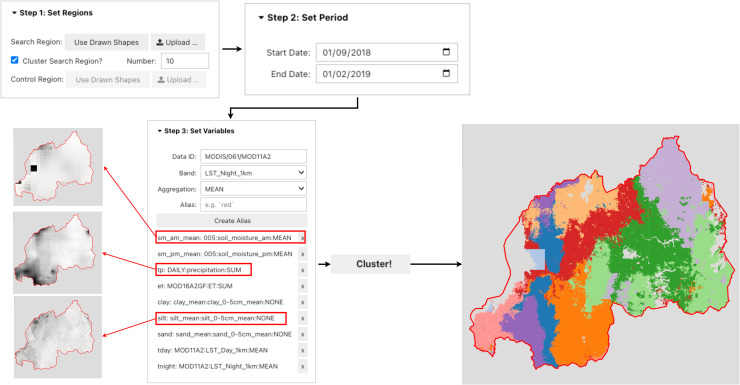
Clustering workflow in Sims. A user uploads a region of interest, sets the number of clusters *k*, the period is defined, selects relevant geospatial variables, and finally runs the clustering algorithm. The resulting clusters group areas with similar geospatial characteristics.

In the clustering workflow (Fig 2), users upload a boundary, choose the period of interest, select geospatial variables from GEE (loaded as descriptive aliases), and set the number of clusters *k*. Sims then (i) temporally aggregates each alias using the chosen reducer (LAST/FIRST/MAX/MIN/MEAN/MEDIAN/SUM/MODE), (ii) clips all layers to the query region, and (optionally) applies a Dynamic World land-cover mask [8]; (iii) standardizes every band via per-band z-score computed over the query region. For model fitting, we draw 5000 samples at 100m resolution, and train GEE’s Weka K-means [9,10] clustering algorithm (ee.Clusterer.wekaKMeans) with user-specified *k*; the distance function defaults to Euclidean (Manhattan is optionally selectable), and all other hyperparameters follow Earth Engine/Weka defaults (e.g., initialization, iteration limits, tolerance, seeding). The trained clusterer is applied to the full feature stack; label IDs are offset to start at 1. Results render in real-time for interactive feedback, and clusters can be exported as rasters—overview maps are reprojected to EPSG:4326 for visualization. No dimensionality reduction or tiling is applied.

### Similarity search

**Fig 3:**
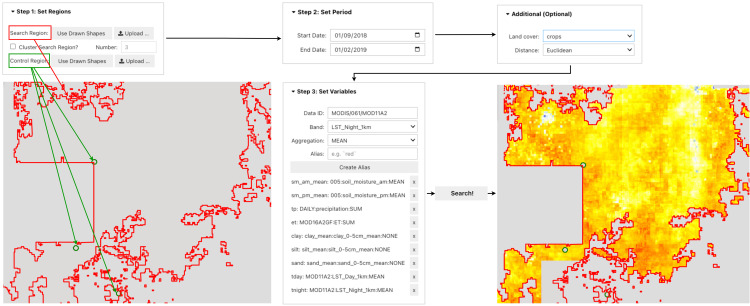
Similarity search workflow in Sims. A user defines the search and reference regions, sets the temporal period, optionally configures land cover masking and distance metrics, selects relevant variables from GEE, and runs the similarity search. The heat map highlights areas sharing similar features with the reference region.

For similarity search (Fig 3), users upload or draw both the search region (where similar areas are sought) and the reference region (the template area). After setting the temporal period and loading relevant geospatial variables, the similarity search algorithm computes the distance (using Euclidean or Cosine metrics) from the reference region to each pixel in the search region. The resulting heat map—possibly refined using land cover masks—can then be exported for downstream analysis.

## Results

### Case study: Maize yield patterns in Rwanda

The Excellence in Agronomy (EiA) initiative of the Consultative Group for International Agricultural Research (CGIAR) addresses global challenges including improving fertilizer access for smallholder farmers in Africa. In Rwanda, for example, the AgWise [11] platform provides targeted fertilizer recommendations for crops such as maize, rice, and potatoes. However, producing large-scale recommendations is challenging, particularly when field observations are sparse.

To demonstrate the potential of Sims, we analyze simulated maize yield data for Rwanda’s January–May cropping seasons between 2005 and 2015 [12]. The aim is to identify distinct yield response zones via clustering of regions using different combinations of spatial variables (soil, weather, and agronomy).

Table 1 shows the feature combinations used for each domain. Detailed alias definitions and feature configurations for these variables are provided in S1 Appendix. For different variable groups and number of clusters (*k*) combinations, we compare the distribution of a medium maturing variety from the simulated maize yield dataset, and assess the significance of the results.

**Table 1:** Features included in each domain for clustering the simulated maize yields in Rwanda.

Feature	Units	Soil	Weather	Agronomy
Total Rainfall	mm		✓	✓
Mean Min/Max Temperature	K		✓	✓
Mean Relative Humidity	%		✓	✓
Mean Evapotranspiration	mm		✓	✓
Clay/Sand Content	g/kg	✓		✓
Nitrogen, Organic Carbon, pH	cg/kg, dg/kg, pH	✓		✓
NDVI	—			✓

**Fig 4:**
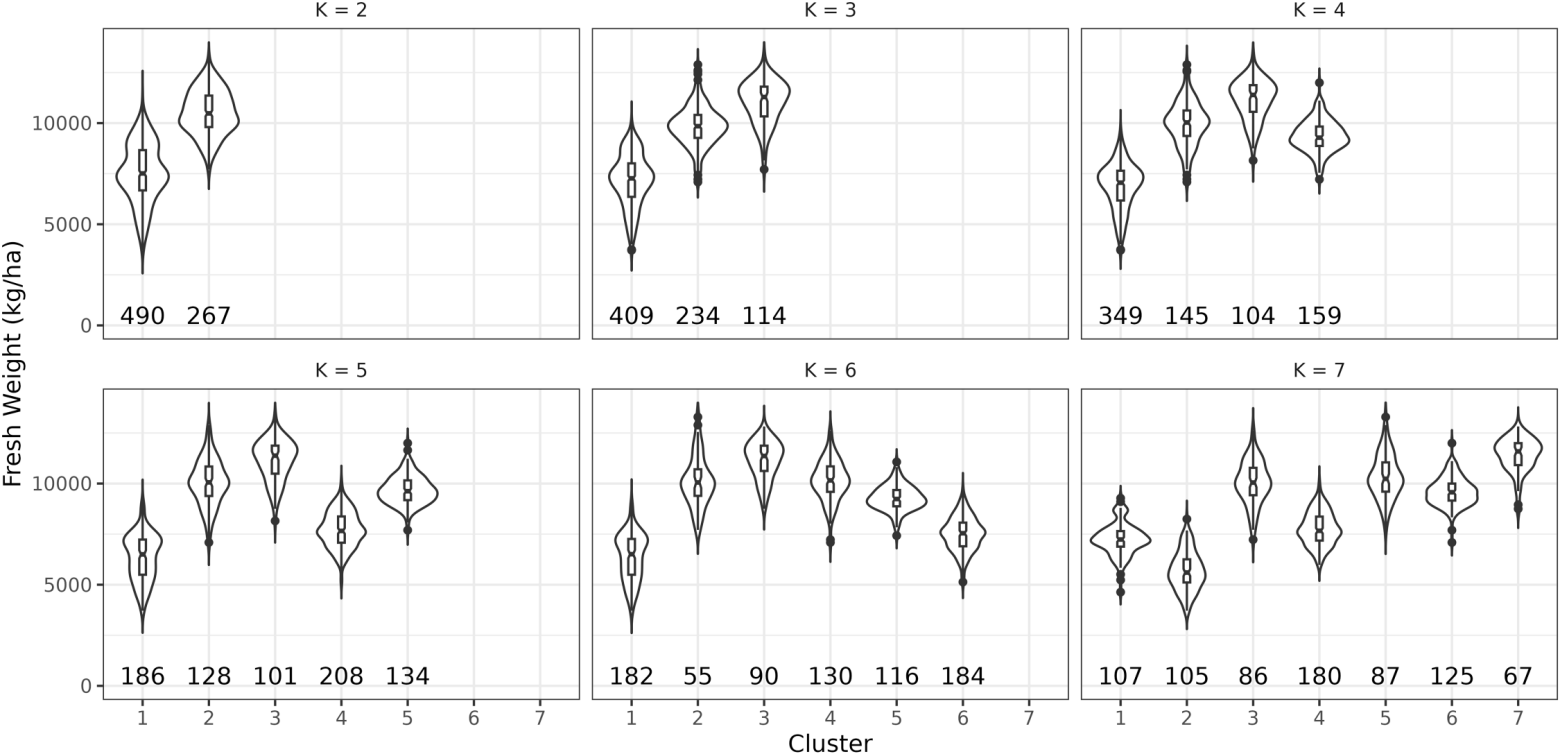
Simulated maize yield (kg/ha) distributions across clusters in Rwanda. Testing different numbers of clusters with Agronomy features revealed highly significant differences ($p<2\times 10^{-16}$) between most cluster pairs at *k* = 5, except for some pairs (e.g., clusters 2 and 5).

Fig 4 shows yield distributions across clustered regions and highlights the cluster combinations that best capture yield variations. We found highly significant differences (p<2e-16) between most cluster pairs at *K* = 5, except clusters 2 and 5 (p≈1) and clusters 2 and 3 (p=1.2e-09). These results demonstrate how users can apply the tool to determine optimal variables and cluster numbers for separating primary maize growing regions.

## Discussion

Sims is designed to be flexible and lightweight. By offloading heavy data computation to GEE, it can run on modest hardware. It supports stacking variables from different data sources, interactive feature exploration, and exporting results for further analysis. In addition to its use in agronomy, Sims offers flexible support for a range of geospatial tasks. For example, in damage mapping, a human-annotated reference region that has been exposed to an extreme event (e.g., flood, drought) can guide the identification of other affected areas. Similarly, urban planners can leverage Sims to cluster neighborhoods based on features such as building density and land use, aiding in targeted infrastructure development. Furthermore, environmental monitoring applications—such as tracking changes in vegetation health or land cover—can utilize Sims to detect early signs of environmental degradation. These use cases underscore the versatility of Sims in addressing diverse challenges across different domains.

### Limitations

Although the interface is intended to be no-code, reaching an operational deployment still demands significant cloud expertise: users must provision Google Cloud resources, configure Earth Engine project permissions, and install local dependencies (or Docker) before any analysis can begin. The current implementation is fully coupled to GEE, offering neither fallback execution for local rasters nor automated pathways to hybridize local datasets with cloud-resident layers. Session state is non-persistent—there is no built-in notion of user authentication, resumable workspaces, or shareable session links beyond exporting static parameter templates—which limits collaborative workflows. Temporal fidelity is constrained because feature layers and distance surfaces are aggregated at a fixed 100 m spatial scale, suppressing short-lived or fine-grained signals. Finally, scalability is bounded by design decisions such as clustering on only 5,000 sampled pixels and exporting results as ∼1 km tiles, which constrains continent-scale or high-resolution campaigns.

## Conclusions and future work

Feature discovery in spatial modeling is often slow because of heavy imagery, expensive data processing, and custom visualization code. Sims addresses this by enabling users to load, transform, and visualize remote sensing layers over regions of interest; it creates similarity and cluster maps that can be exported for further analysis.

One promising future work direction that will broaden access beyond browser-based environments is to create a QGIS plugin that reuses the core Sims logic while integrating with QGIS’ native UI, data stack, and processing backends. QGIS already provides raster and vector layer editing and aliases; these could be mapped to Sims’ alias system. Only thin panels would be needed for GEE catalog search and controls (region/period selection, number of clusters, clustering method, export). Such a plugin could store the user’s GEE credentials to authenticate requests, retrieve data, and submit server-side clustering and similarity jobs; returned assets could be added to the project as standard QGIS raster/vector layers for immediate interaction. Local data could also be supported: users may upload local rasters to GEE to run cloud workflows, or bypass GEE entirely and operate on local GeoTIFF/COG rasters and GeoParquet/GeoPackage/GeoJSON/Shapefile vectors using GDAL-backed operators—particularly fast for small ROIs. Large-area or catalog-scale analyses could dispatch to GEE (image collections and reducers).

## Supporting information

S1 Appendix Alias definitions and feature configurations used in the Rwanda case study. (PDF)
