# Supplementary material for: Sims: An interactive tool for geospatial matching and clustering
Source: PLoS One. 2026 Apr 8;21(4):e0344525. doi: 10.1371/journal.pone.0344525 (PMC13061242; doi:10.1371/journal.pone.0344525)
Supplement: S1 Appendix — (PDF) [file pone.0344525.s001.pdf]

Appendix: Aliases & Features Used in the Clustering Case Study

0.1 Soil Aliases & Features Used in the Clustering Case Study

Aliases within Sims are defined in the format:

> *{alias_name}:{Product_ID}:{Product_band}:*
> *{Start_date}:{End_date}:{Aggregation_function}*

- **alias_name**: A short, descriptive name for the variable, making it easy to reference within the tool. Created by the user.
- **Product_ID**: The identifier for the specific dataset within GEE.
- **Product_band**: The specific band within the dataset that contains the data of interest, such as soil moisture or temperature.
- **Start/End_Date**: Defines the period of interest.
- **Aggregation_function**: Applied to aggregate an image collection over the defined temporal domain, such as MEAN, SUM, or LAST. Produces a single image.

0.1.1 Aliases

- **Soil Clay**
  - `clay5:soilgrids-isric/clay_Mean:`
    `clay_0-5cm_Mean:`
    `01/01/2010:31/12/2020:LAST`
  - `clay15:soilgrids-isric/clay_Mean:`
    `clay_5-15cm_Mean:`
    `01/01/2010:31/12/2020:LAST`
  - `...`
  - `clay100:soilgrids-isric/clay_Mean:`
    `clay_60-100cm_Mean:`
    `01/01/2010:31/12/2020:LAST`
- **Soil Sand**
  - `sand5:soilgrids-isric/sand_Mean:`
    `sand_0-5cm_Mean:`
    `01/01/2010:31/12/2020:LAST`
  - `sand15:soilgrids-isric/sand_Mean:`
    `sand_5-15cm_Mean:`
    `01/01/2010:31/12/2020:LAST`
  - `...`
  - `sand100:soilgrids-isric/sand_Mean:`
    `sand_60-100cm_Mean:`
    `01/01/2010:31/12/2020:LAST`
- **Soil Organic Carbon**
  - `soc5:soilgrids-isric/ocd_Mean:`
    `ocd_0-5cm_Mean:`
    `01/01/2010:31/12/2020:LAST`

- soc15:soilgrids-isric/ocd_Mean:
  ocd_5-15cm_Mean:
  01/01/2010:31/12/2020:LAST
- ...
- soc100:soilgrids-isric/ocd_Mean:
  ocd_60-100cm_Mean:
  01/01/2010:31/12/2020:LAST

- **Soil Total Nitrogen**
  - n5:soilgrids-isric/nitrogen_Mean:
    nitrogen_0-5cm_Mean:
    01/01/2010:31/12/2020:LAST
  - n15:soilgrids-isric/nitrogen_Mean:
    nitrogen_5-15cm_Mean:
    01/01/2010:31/12/2020:LAST
  - ...
  - n100:soilgrids-isric/nitrogen_Mean:
    nitrogen_60-100cm_Mean:
    01/01/2010:31/12/2020:LAST
- **Soil pH H2O**
  - ph5:soilgrids-isric/phh2o_Mean:
    phh2o_0-5cm_Mean:
    01/01/2010:31/12/2020:LAST
  - ph15:soilgrids-isric/phh2o_Mean:
    phh2o_5-15cm_Mean:
    01/01/2010:31/12/2020:LAST
  - ...
  - ph100:soilgrids-isric/phh2o_Mean:
    phh2o_60-100cm_Mean:
    01/01/2010:31/12/2020:LAST

0.1.2 Features

- clay:MEAN(clay*)
- sand:MEAN(sand*)
- soc:MEAN(soc*)
- ntot:MEAN(n*)
- ph:MEAN(ph*)

0.2 Weather Aliases & Features Used in the Clustering Case Study

0.2.1 Aliases

- **Rainfall**

  - rain05:UCSB-CHG/CHIRPS/DAILY: precipitation: 01/12/2004:31/07/2005:SUM
  - rain06:UCSB-CHG/CHIRPS/DAILY: precipitation: 01/12/2005:31/07/2006:SUM
  - ...
  - rain15:UCSB-CHG/CHIRPS/DAILY: precipitation: 01/12/2014:31/07/2015:SUM

- **Max Temperature**
  - tmax05:MODIS/061/MOD11A2: LST_Day_1km: 01/12/2004:31/07/2005:MAX
  - tmax06:MODIS/061/MOD11A2: LST_Day_1km: 01/12/2005:31/07/2006:MAX
  - ...
  - tmax15:MODIS/061/MOD11A2: LST_Day_1km: 01/12/2014:31/07/2015:MAX

- **Min Temperature**
  - tmin05:MODIS/061/MOD11A2: LST_Night_1km: 01/12/2004:31/07/2005:MIN
  - tmin06:MODIS/061/MOD11A2: LST_Night_1km: 01/12/2005:31/07/2006:MIN
  - ...
  - tmin15:MODIS/061/MOD11A2: LST_Night_1km: 01/12/2014:31/07/2015:MIN

- **Relative Humidity**
  - rhum05:UCSB-CHG/CHIRTS/DAILY: relative_humidity: 01/12/2004:31/07/2005:MEAN
  - rhum06:UCSB-CHG/CHIRTS/DAILY: relative_humidity: 01/12/2005:31/07/2006:MEAN
  - ...
  - rhum15:UCSB-CHG/CHIRTS/DAILY: relative_humidity: 01/12/2014:31/07/2015:MEAN

- **Evapotranspiration**

  - et10:FAO/WAPOR/2/L1_AETI_D:
    L1_AETI_D:
    01/12/2009:31/07/2010:MEAN
  - et11:FAO/WAPOR/2/L1_AETI_D:
    L1_AETI_D:
    01/12/2010:31/07/2011:MEAN
  - ...
  - et15:FAO/WAPOR/2/L1_AETI_D:
    L1_AETI_D:
    01/12/2014:31/07/2015:MEAN

0.2.2 Features

- rain:MEAN(rain*)
- tmax:MEAN(tmax*)
- tmin:MEAN(tmin*)
- rhum:MEAN(rhum*)
- et:MEAN(et*)

0.3 Agronomy Aliases & Features Used in the Clustering Case Study

0.3.1 Aliases

- **NDVI**
  - ndvi05:MODIS/061/MOD13A2:
    NDVI:
    01/12/2004:31/07/2005:MEAN
  - ndvi06:MODIS/061/MOD13A2:
    NDVI:
    01/12/2005:31/07/2006:MEAN
  - ...
  - ndvi15:MODIS/061/MOD13A2:
    NDVI:
    01/12/2014:31/07/2015:MEAN

0.3.2 Features

- ndvi:MEAN(ndvi*)
